# Multiple system atrophy: genetic risks and alpha-synuclein mutations

**DOI:** 10.12688/f1000research.12193.1

**Published:** 2017-11-30

**Authors:** Heather T Whittaker, Yichen Qui, Conceição Bettencourt, Henry Houlden

**Affiliations:** 1 Department of Molecular Neuroscience, UCL Institute of Neurology, London, UK; 2 Department of Clinical and Experimental Epilepsy, UCL Institute of Neurology, London, UK; 3 MRC Centre for Neuromuscular Diseases, UCL Institute of Neurology, London, UK; 4 Neurogenetics Laboratory, The National Hospital for Neurology and Neurosurgery, London, UK

**Keywords:** multiple system atrophy, MSA, neurodegenerative disorders, α-synuclein

## Abstract

Multiple system atrophy (MSA) is one of the few neurodegenerative disorders where we have a significant understanding of the clinical and pathological manifestations but where the aetiology remains almost completely unknown. Research to overcome this hurdle is gaining momentum through international research collaboration and a series of genetic and molecular discoveries in the last few years, which have advanced our knowledge of this rare synucleinopathy. In MSA, the discovery of α-synuclein pathology and glial cytoplasmic inclusions remain the most significant findings. Families with certain types of α-synuclein mutations develop diseases that mimic MSA, and the spectrum of clinical and pathological features in these families suggests a spectrum of severity, from late-onset Parkinson’s disease to MSA. Nonetheless, controversies persist, such as the role of common α-synuclein variants in MSA and whether this disorder shares a common mechanism of spreading pathology with other protein misfolding neurodegenerative diseases. Here, we review these issues, specifically focusing on α-synuclein mutations.

## Introduction

Multiple system atrophy (MSA) is a neurodegenerative movement disorder affecting around 1 in 20,000 people ^1, 2^. It occurs sporadically, usually presenting between the age of 35 and 65 years with a variable combination of parkinsonian, cerebellar, and autonomic features and rapidly progressing for 9 years on average ^3– 6^. According to the presenting clinical features and predominant manifestations, MSA is usually categorised as MSA-C or MSA-P, there can be mixed signs, and some patients present with autonomic features. Familial MSA has been reported but in only a handful of cases worldwide. Research toward potential treatments for MSA, as with many rare diseases, has been limited, resulting in a paucity of knowledge regarding its underlying causes. Initial clues came from studying α-synuclein (SNCA) and the hallmark histopathology in the brains of patients with MSA: glial cytoplasmic inclusions (GCIs) that reside predominantly in oligodendrocytes, the post mortem identification of which is required for a definitive diagnosis. Besides MSA, the only conditions that have GCIs in the brain are certain families with SNCA mutations. Three groups found that the GCIs contain abnormal forms of SNCA protein ^7– 12^, the same protein that accumulates in Parkinson’s disease (PD) and dementia with Lewy bodies ^12^. These studies were motivated by a link between point mutations in the *SNCA* gene and heritable forms of PD ^13, 14^. The similarities between MSA and PD have proven more complicated to disentangle, as SNCA mutations in some families clinically and pathologically resemble MSA and others even have features of frontal dementia with severe pathology ^15^.

It has been nearly two decades since MSA was characterised as a synucleinopathy, and apart from the PD-MSA overlap identified in SNCA families, researchers have not been able to further understand the aetiology of MSA or alter or halt the disease process. This brief review will set out the progress that has been made in recent years toward understanding the pathomechanisms of SNCA aggregation and toxicity in relation to MSA, particularly the emerging hypotheses of aetiology based on genetic studies. As clinical trials targeting SNCA proceed in PD and MSA, there is increasing urgency to better understand its relevant cellular interactions in parallel with the development of sensitive biomarkers capable of diagnosing patients at an earlier disease stage.

## The role of α-synuclein in multiple system atrophy

Despite the key involvement of abnormal SNCA processing, misfolding, and aggregation in synucleinopathies, the normal function of the protein is not fully understood. It is a peripheral membrane protein, localized at nerve terminals where it is thought to play a role in the release of neurotransmitters, and recently has been reported to enhance transient synaptic vesicle fusion ^16, 17^ and possibly disrupt the support and maintenance of neurons provided by oligodendrocytes. In MSA, SNCA is deposited widely, but there are more severely affected regions such as the basal ganglia, cerebellum, pons, inferior olivary nuclei, and spinal cord ^18, 19^. Not only is SNCA deposition clearly distinguishable between MSA and PD cases but MSA-like pathology underlies both cerebellar (MSA-C) and parkinsonian (MSA-P) manifestations. There is also minimal change MSA pathology in some cases that have a longer disease duration. How this single protein can apparently be the culprit in these different disease phenotypes, with such varied localization in different cell types and brain regions, is an unresolved question.

One compelling explanation for the clinicopathological diversity in the synucleinopathies is that distinct strains of SNCA are responsible for generating heterogeneity ^20^. These conformational variants include different oligomer combinations, fibrils and ribbons, although their relative contribution to the anatomical distribution and deposition of SNCA in MSA and other synucleinopathies and the formation of GCIs in MSA has yet to be determined. Furthermore, it has been posited that the specific structure of SNCA derived from inclusions in the brains of patients with MSA is especially toxic, capable of propagating to adjacent cells and inducing neurodegeneration when injected into transgenic mice, akin to the permissive templating of prion protein and even prompting reclassification of MSA as a prion disease ^21, 22^. However, it remains to be shown conclusively that oligodendroglial MSA-type pathology is provoked by seeded aggregation of SNCA.

The pathomechanisms of MSA are being steadily elucidated as studies examine the molecular interactions of SNCA with other proteins in MSA. A recent study ^23^ has reported that SNCA engages with proteins that regulate autophagy in the MSA brain, implicating cellular degradation as central to the pathogenesis of MSA and potentially unifying it with other neurodegenerative diseases for the purpose of therapeutic intervention of these pathways ^23, 24^. Additionally, there is an emerging conviction that SNCA induces deficits in myelination ^25^ and there is a possible role for inflammatory/apoptotic mechanisms.

## Mutations and copy number variation in α-synuclein

**Figure 1.**
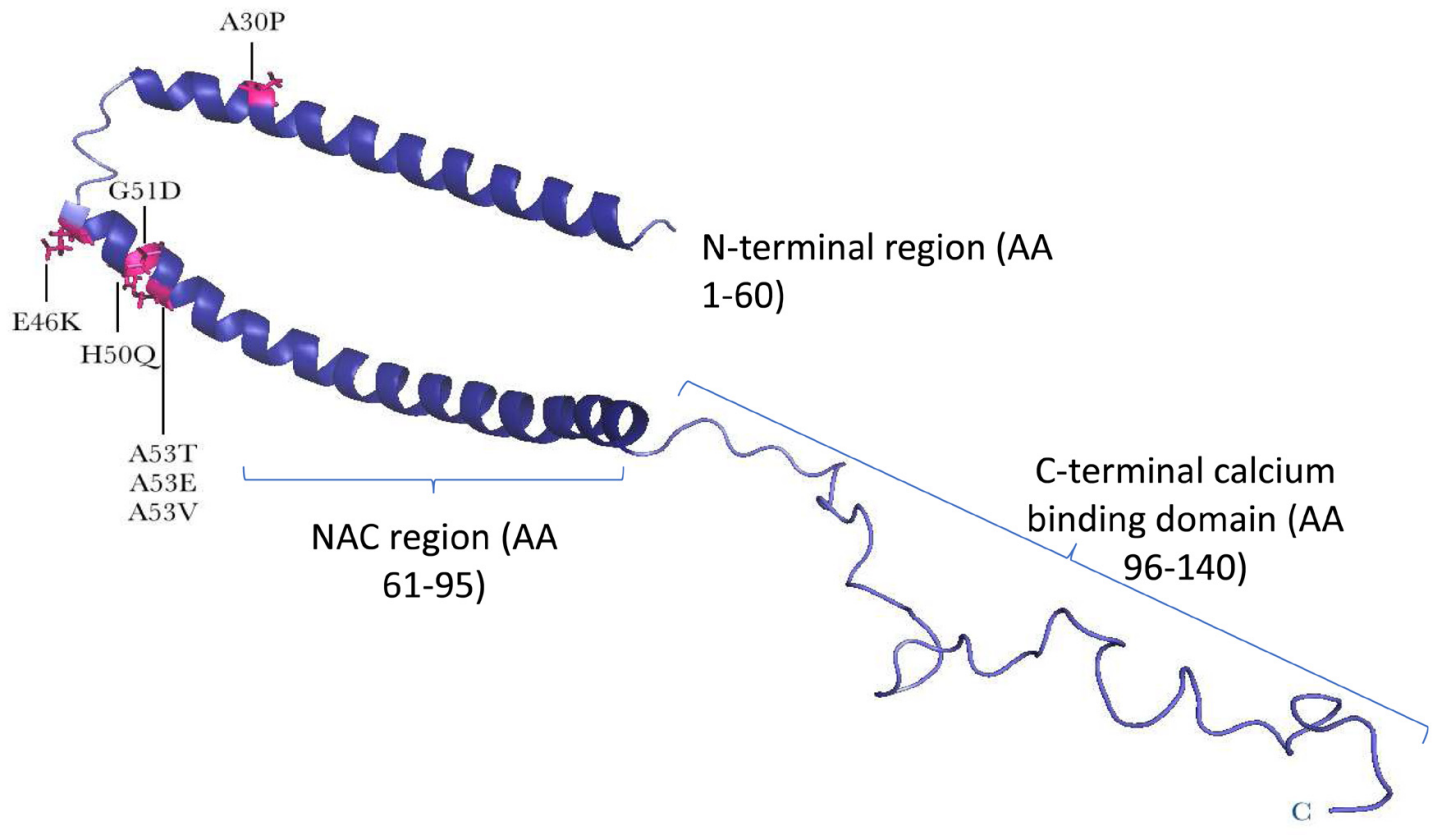
Structural features of the alpha-synuclein monomer. A structure of the full-length, membrane-bound form of alpha-synuclein (SNCA) protein reveals a conformation in which the N-terminal two-thirds of the protein forms a broken, amphipathic alpha-helix. This structured portion of the protein is responsible for membrane binding, and residues at the very N-terminus are essential for this process. In the nuclear magnetic resonance structure of SNCA, the negatively charged C-terminal tail remains flexible and disordered (based on Yu *et al*. ^46^). The positions of point mutations associated with Parkinson’s disease are indicated with arrows and in pink. All mutations are heterozygous, except for p.A53V, which is homozygous.

The initial genome-wide association study (GWAS) in PD yielded significant association at the *SNCA* and microtubule-associated protein tau ( *MAPT*) genes ^26^. Common variation in the gene encoding SNCA was first identified as a risk factor for MSA in 2009 ^27^, but the association of variants across *SNCA* in different populations was not replicated in later studies ^28– 32^ and was thought to be due to a mixed control population used in the initial studies. The first GWAS to be conducted in MSA yielded negative results around the *SNCA* locus ^28^. As mentioned earlier, several SNCA point mutations ^14, 33– 39^ and *SNCA* gene duplications ^40^, triplications ^41, 42^, and double duplications ^43^ have been associated with familial forms of PD ( Figure 1 and Table 1 and Table 2). Some of these families have manifestations of both PD and MSA and have clinical signs or neuropathological features or both ^44, 45^. In particular, the A53T, A53E, and G51D mutations and *SNCA* gene triplications are associated with a more aggressive MSA-like clinical and pathological phenotype ^45^ (See Table 1 and Table 2 for details of the clinical and neuropathological features of SNCA mutations). Exactly why the codon 51 and 53 mutations in the *SNCA* gene lead to an MSA-like clinical and pathological phenotype is not known, but this is likely to be associated with the importance of this defined region and toxic gain of function of these protein changes ( Figure 1) ^46^. From a clinical perspective, if there is any hint of a family history in patients with MSA, then the *SNCA* gene should be sequenced by using traditional Sanger ^47^, gene panel, or exome sequencing and analysed for copy number changes ^48^.

**Table 1.** Clinical features of families with alpha-synuclein (SNCA) mutations.

SNCA protein change	p.Ala30Pro ^1^	p.Glu46Lys ^2^	p.His50Gln ^3^	p.Gly51Asp ^4, 5^	p.Ala53Thr ^6^	p.Ala53Glu ^7^	p.Ala53Val ^8^	Duplication ^9^	Triplication ^10^	Double duplication ^11^
*SNCA*: 5′ start - ATG start -	*177G>C* c.88G>C	*225G>A* c.136G>A	*239T>G* c.150T>G12	*241G>A* c.152 G>A	*246G>A* c.157G>A	*247C>A* c.158C>A	*247C>T* c.158C>T	Whole gene copy number	Whole gene copy number	Whole gene copy number
Zygosity	Heterozygous	Heterozygous	Heterozygous	Heterozygous	Heterozygous	Heterozygous	Homozygous/ heterozygous	Gene duplication on one allele	Gene triplication on one allele	Gene duplication on both alleles
Clinical phenotype	Classic PD	Dementia with Lewy bodies phenotype	Classic PD	Severe PD with some patients with MSA features	Severe PD with some patients with MSA features	Severe PD with some patients with MSA features	Homozygous = PD Heterozygous = cognitive decline or psychosis	Usually classic PD, some with severe cognitive and frontal dementia ^13^	Severe PD with some patients with MSA features	Severe PD
Family	German ^14^	Spanish Basque Country ^15^	English ^16^	British ^4^, French ^17^, and Japanese ^18^	Large Sicilian (Contursi) kindred ^6^ and Greek ^19^, Swedish ^20^, Korean ^21^	Finnish ^22^	Japanese ^23^	French ^24^, Italian ^25^, Japanese ^26, 27^, Korean ^28^, Swedish ^29^, UK, Welsh ^13^	Spellman- Muenter (Iowa) kindred ^30^, Swedish ^9^	Pakistani ^31^
Estimated penetrance/ risk	71.4%	30%	30% Heterozygous ExAc =4/121,306	100%	85%	100%	Homozygous 100% Heterozygous on ExAc = 1/121,304	44% ^27^	100%	100%
Mean age of onset, years	60	50–65	71	44 (as early as 19) ^5^	48	43	59	50	40	31
Clinical symptoms	Progressive parkinsonism, walking difficulties, no other non-motor symptoms except cognitive decline (50% of patients)	Resting tremor, bradykinesia, postural instability, severe immobility, dementia, and visual hallucinations	Resting hand tremor, benign course	Resting tremor, dystonia, cognitive/frontal decline, anxiety, depression, visual hallucination, and autonomic disturbances ^5^	Moderate tremor, rigidity, bradykinesia, postural instability, severe dementia, depression, and autonomic disturbance ^32^	Bradykinesia, resting tremor, rigidity, insomnia, spasticity, myoclonic jerks, anxiety, and panic disorders	Bradykinesia, resting tremor, rigidity, mild cognitive decline, visual hallucination, sleep disorder, delusions, and paranoia	Bradykinesia, resting tremor, rigidity, mild asymmetric onset. Some have epilepsy, depression, and frontal dementia.	Severe early onset parkinsonism, resting tremor, bradykinesia, rigidity, and symptoms of MSA	Severe bilateral bradykinesia rigidity, mild resting tremor, severe depression, and postpartum psychosis
Sustained response to levodopa	Transient marked, developed hallucinations	Mild to moderate, developed hallucinations and conscious fluctuation	Moderate	Transient marked, developed choreiform movements	Marked	Marked, developed dyskinesia ^33^	Marked	Mild to moderate, developed motor fluctuation and dyskinesia	Marked	N/A

ExAC, http://exac.broadinstitute.org; MSA, multiple system atrophy; N/A, no data available; PD, Parkinson’s disease; *SNCA*, alpha-synuclein gene.

**Table 2.** Neuropathological features of families with alpha-synuclein (SNCA) mutations.

SNCA protein change	p.Ala30Pro ^1^	p.Glu46Lys ^2^	p.His50Gln ^3^	p.Gly51Asp ^4, 5^	p.Ala53Thr ^6, 34^	p.Ala53Glu ^7^	Duplication ^35^	Triplication ^10^
Neuropathology summary	Widespread synuclein neuropathology and LBs	Widespread synuclein and ubiquitin neuropathology and LBs	Widespread synuclein neuropathology and LBs	Widespread synuclein neuropathology, LBs, and GCIs	Widespread synuclein neuropathology, LBs, and GCIs	Widespread synuclein neuropathology, LBs, and GCIs	Widespread synuclein neuropathology, LBs, and some GCIs	Widespread synuclein neuropathology, LBs, and GCIs
LBs	Yes	Yes	Yes	Yes	Yes	Yes	Yes	Yes
Cortical neuronal loss	Widespread	Widespread	Not identified	Widespread, moderate to severe, anterior temporal, piriform, and insular cortices ^4^	Widespread, moderate to severe, mid- frontal cortex, superior temporal cortex, and inferior parietal cortex	Widespread and severe	Not identified	Widespread
Hippocampal neuronal loss	CA1/2/3	Not identified	Not identified	Severe in CA2/3 ^4^	Severe in CA1, moderate in CA2/3 ^34^	Mild in CA1/2/3	CA2/3 region	CA2/3 region
Brain stem neuronal loss	Substantia nigra, locus coeruleus, and dorsal nuclei of the vagus	Substantia nigra, locus coeruleus, and dorsal nuclei of the vagus	Substantia nigra	Substantia nigra, locus coeruleus, and dorsal motor nuclei of vagus	Substantia nigra and locus coeruleus	Substantia nigra	Substantia nigra, locus coeruleus, and dorsal motor nuclei of vagus	Substantia nigra and locus coeruleus
Neuronal α-synuclein pathology	Globular, spherical, reniform, widespread om cortex and brainstem	Concentric, nonconcentric, granular	PD-type (Braak stage 6)	Annular, crescentic, globular, diffuse, NFT-like, Widespread cortical	Wide spread cortex and brain stem, LBs	Annular, annular, LB-like inclusions, mild in brainstem	PD-type (Braak stage 6), widespread from brainstem and neocortex	Widespread in cortex, few in brainstem
Glial α-synuclein pathology	No	No	No	GCI-like	GCI-like	Granular GCI	GCI-like	Atypical LBs, GCIs
Phosphorylated tau Braak and Braak stage	II	Not identified	III	IIa	I	N/A	I	N/A
Aβ deposition	Thal phase 1 ^36^	Neocortical	Neocortical	N/A	N/A	N/A	Sparse neocortical	N/A

There has been no neuropathology on the p.Ala53Val mutation, and the double duplication has no brain donation. GCI, glial cytoplasmic inclusions; LB, Lewy body; N/A, no data available; NFT, neurofibrillary tangle; PD, Parkinson’s disease.

## Other genetic risk factors for multiple system atrophy

A number of PD risk factors have not been replicated in MSA ^4, 6, 48– 61^, but other disorders such as spinocerebellar ataxia type 17 and progressive supranuclear palsy ^62– 64^ can mimic MSA in the early stages and should be included in clinical and genetic testing. In a statistical analysis of 5,302 patients with PD and 4,161 controls from 15 sites, Elbaz and colleagues found no evidence for an interactive effect between the H1 haplotype in the *MAPT* gene and single-nucleotide polymorphisms in the *SNCA* gene on disease ^65^. Variation in each gene was associated with PD risk, indicating independent effects. In MSA, the H1 haplotype has been associated with MSA ^66^ and the *MAPT* gene was also implicated in the MSA GWAS ^28^. Familial inheritance of MSA is rare but has been observed. These families often have atypical clinical features, and the genetic analysis led to the discovery of mutations in the *COQ _2_* gene, which plays a role in synthesising the mitochondrial electron transporter and antioxidant coenzyme Q _10_. These mutations were posited to impair the activity of the mitochondrial respiratory chain and increase oxidative stress, implicating COQ _2_ variants as a risk factor for sporadic MSA ^58^. Though initially promising, these findings have not been consistently replicated in various populations, refuting COQ _2_ polymorphisms as common MSA risk factors ^58^. Nonetheless, this has turned attention, and emerging hypotheses centre on mitochondrial dysfunction as a central component of the pathophysiological cascade in MSA ^67^.

The first GWAS in MSA was carried out by Sailer and colleagues and was extremely important but challenging given the rarity of MSA ^28^. At just under 1,000 MSA cases, the analysis was still statistically under-powered ^28^. Studies that are more highly powered are needed to follow up on the importance of the three genes identified that were flagged for being associated: *FBXO47*, *ELOVL7*, and *MAPT* ^28^. It will be important to follow this GWAS up with greater numbers of MSA cases, analyse age at onset association ^68– 70^, and employ advanced transcriptome sequencing in MSA patient brain tissue to assess the associated genes and other genes thought to be involved in MSA, such as immune-responsive and iron metabolism genes ^71, 72^.

## Clinical genetic testing and translation

Accurate and early diagnosis of MSA continues to be an important research objective as the heterogeneous features of PD and other atypical parkinsonism syndromes can mimic MSA. One retrospective clinicopathological study revealed that 38% of patients were misdiagnosed with MSA on the basis of expert interpretation of their symptomatic presentation ^73– 75^. Genetic analysis will be important to identify the rare MSA cases with *SNCA* mutations and to help differentiate MSA from similar disorders such as spinocerebellar ataxia type 17 ^62, 64^. Thus, biomarkers that are more sensitive are imperative to improve diagnosis and enlist individuals with the appropriate disease in clinical trials. This will be imperative in the development of effective treatments for the MSA patient population. Both α-synuclein and CoQ _10_ are being pursued as potential therapeutic targets, and international collaborative study groups are promoting this work with CoQ _10_ supplementation, the preparation of α-synuclein antisense oligonucleotide, and immunisation trials to be conducted in PD and MSA patients by either intravenous or intrathecal routes.

Until disease-modifying treatments become available, symptom management will remain the mainstay of care for patients with MSA. Patient support organisations such as the MSA Trust ( www.msatrust.org.uk/) and the MSA coalition ( https://www.multiplesystematrophy.org/) and their clinical nurse specialists are essential in providing support and advice on patient care in this rare disorder. The established drugs for controlling parkinsonism, such as L-dopa, can be effective in the early stages of MSA but often worsen the symptoms due to hypotension later in the disease. A rational treatment, based on the pathophysiology of MSA and perhaps repurposed from PD trials, needs to be developed to offer patients with MSA hope for this devastating disorder.
